# Patients’ and healthcare workers’ recommendations for a surgical patient safety checklist – a qualitative study

**DOI:** 10.1186/s12913-020-4888-1

**Published:** 2020-01-16

**Authors:** Kristin Harris, Eirik Søfteland, Asgjerd Litleré Moi, Stig Harthug, Anette Storesund, Sebastius Jesuthasan, Nick Sevdalis, Arvid Steinar Haugen

**Affiliations:** 1 Department of Health and Caring Sciences, Western Norway University of Applied Sciences, Inndalsveien 28, 5063 Kronstad, Bergen, Norway; 2 Department of Anesthesia and Intensive Care, Haukeland University Hospital, Postboks 1400, 5021 Bergen, Norway; 3 Department of Clinical Medicine, University of Bergen, Postboks 7804, 5020 Bergen, Norway; 4 Department of Plastic, Hand and Reconstructive Surgery, National Burn Centre, Haukeland University Hospital, Postboks 1400, 5021 Bergen, Norway; 5 Department of Research and Development, Haukeland University Hospital, Postboks 1400, 5021 Bergen, Norway; 6 Department of Clinical Science, University of Bergen, Postboks 7804, 5020 Bergen, Norway; 7 Department of Surgery, Førde Comunity Hospital, Postboks 1000, 6807 Førde, Norway; 8 Centre for Implementation Science, Health Service & Population Research Department, King’s College,16 De Crespigny Park, London, UK SE5 8AF UK

**Keywords:** Patient’s surgical safety checklist, Patient surgical safety, Patient involvement, Patient surgical information

## Abstract

**Background:**

Patients’ involvement in patient safety has increased in healthcare. Use of checklists may improve patient outcome in surgery, though few have attempted to engage patients’ use of surgical checklist. To identify risk elements of complications based on patients’ and healthcare workers’ experiences is warranted. This study aims to identify what the patients and healthcare workers find to be the risk elements that should be included in a patient-driven surgical patient safety checklist.

**Method:**

A qualitative study design where post-operative patients, surgeons, ward physicians, ward nurses, and secretaries from five surgical specialties took part in focus group interviews. Eleven focus groups were conducted including 25 post-operative patients and 27 healthcare workers at one tertiary teaching hospital and one community hospital in Norway. Based on their experiences, participants were asked to identify perceived risks before and after surgery. The interviews were analysed using content analysis.

**Results:**

Safety risk factors were categorised as pre-operative information: pre-operative preparations, post-operative information, post-operative plans and follow-up. The subcategories under pre-operative information and preparations were: contact information, medication safety, health status, optimising health, dental status, read information, preparation two weeks before surgery, inform your surgical ward, planning your own discharge, preparation on admission and just before surgery. The subcategories under post-operative information, further plans and follow-up were: prevention and complications, restriction and activity, medication safety, pain relief, stomach functions, further care and appointments. Both healthcare workers and patients express the need for a surgical patient safety checklist.

**Conclusion:**

A broad spectre of risk elements for a patient safety checklist were identified. Developing a surgical safety checklist based on these risk elements might reduce complications and unwanted errors.

**Trail registration:**

The study is registered as part of a clinical trial in ClinicalTrials.gov: NCT03105713.

## Background

The World Health Organisation (WHO) and European patient organisations endorse patient involvement in safety [1, 2]. Patient involvement in self-care and safety is widely discussed [1, 3–7]. Patients are willing to participate, but this depends largely on the healthcare system creating opportunities that promote and allow patient involvement [2, 5, 8]. There are decision aids increasing patients’ involvement in treatment processes by enhancing their knowledge of risks and benefits related to specific treatments [9, 10].

Systematic literature reviews on patient involvement in safety show that patients have an important role on their own safety, but evidence on effects of such involvement is limited [3, 11, 12]. There have been numerous attempts to facilitate use of patient-centred checklist pamphlets and apps [13–16]. These tools are often not aligned with different hospital administrative systems and the patients’ medical records, limiting their potential as communication tools to prevent errors [17]. Several key elements that could potentially prevent medical and surgical complications, such as empowering the patients to request information, but also informing them on the importance of optimising their own health (e.g., before and after undergoing surgical procedures), have been identified. However, such interventions need to be initiated at the right time with the right tools to be effective [18].

Prehabilitation and Enhanced Recovery after Surgery (ERAS) is a multidisciplinary and multimodal perioperative program designed to optimise patient health before surgery, increase awareness in certain elements within the intraoperative phase as well as enhancing patient recovery and rehabilitation. With patient compliance, these programs have shown reductions up to 50% in complications, and 42% in mortality in patients [7, 19]. Further, one study having designed and implemented a patient checklist after colorectal surgery showed reduction of readmissions from 28% to 20% [16]. These findings might indicate that introducing a patient checklist before and after surgery can inform patients better about what they can do to prevent complications and enhance their safety knowledge.

Surgical checklists driven by healthcare providers have flourished within surgical care in the last decade. Checklists, such as the WHO Surgical Safety Checklist in operating theatres and the comprehensive Surgical Patient Safety System (SURPASS) throughout the surgical pathway, have been shown to improve patient safety by preventing medical errors and to reduce morbidity and mortality [20–23]. How patients can be involved in applying checklists has been recommended, but has not been significantly explored [3, 24]. A checklist for patients to use might enhance patient – physician communication, increase patient’s participation in their own safety and optimise patient health [3, 6, 24].

Little is currently known about patient-driven checklist in surgery. It remains unknown whether patients would be comfortable and able to use a checklist as part of their own care. More importantly, which patient safety elements should be included in a checklist and which of these the patients themselves perceive as important, lacks investigation. Based on patients’ and healthcare workers’ experiences, this study aims to explore and describe the risk elements and perceived content for a safety checklist to be used before and after surgery.

## Methods

The study has an exploratory qualitative study design, with focus group interviews involving healthcare workers and discharged surgical patients to gain patients’ and healthcare workers’ experiences and perspectives on preventable risk factors throughout the surgical pathway. When designing the study, patient representatives from the Health Trust Patient Advisory Board were consulted for their opinion on the study design and interview guide. In addition, we followed the Consolidated criteria for Reporting Qualitative Research (COREQ) checklist in reporting our research.

### Settings and participants

Participants were recruited from surgical wards at two Norwegian hospitals, one tertiary teaching hospital and one community hospital, being referrals for 1.1 million and 110,000 inhabitants, respectively. The surgical specialties included were: Ear, Neck, Throat (ENT)−/Maxillo-Facial-; Cardio-Thoracic-; Neuro-; Breast- and Endocrine-; and General surgery. Eligible elective surgical patients from the participating wards were aged 18 years or older, without mental health conditions, independent in daily life and living within one hour’s drive from the hospital, were asked if they were willing to participate in the study. Potential participants were recruited in collaboration with the ward nurses and there was no prior relationships with researchers before study start. Service managers recruited healthcare workers (surgeons, ward physicians, ward nurses, and secretaries) strategically, based on experience and type of profession, and the interviews were conducted within workhours. No quality or risk managers of the clinics participated in the focus groups interviews because they were involved with the project*.* One or two of each profession mentioned above participated in each health care workers’ focus group interview. Five focus groups interviews of surgical healthcare workers and six groups of surgical patients were conducted with five to eight participants per group (25 patients and 27 healthcare workers, in total; participants’ demographic data are presented in Table 1). One additional patient focus group interview was conducted due to too few participants in one of the groups. The patients were recruited one to two days before hospital discharge and interviewed three to six weeks after discharge.

**Table 1:** Patients (*n* = 25) and healthcare personnel (*n* = 27) characteristics

	Age in years	Sex	Professional experience in years
Interview participants	Mean (SD)	Male n (%)	Mean (SD)
Healthcare personnel	43.0 (10.7)	7 (25.9%)	12.4 (9.3)
Patients	53.9 (15.1)	8 (32.0%)	–
Abbreviations: *SD* Standard deviation			

### Data collection

The interviews were conducted from February to June 2017. Two separate semi-structured interview guides were used, one for healthcare workers and one for patients. The interview guides were developed based on earlier research on safety checklists [3, 21, 25, 26]. Each interview guide was piloted in separate healthcare workers and patient focus group interviews. The interview guides were similar, but each adapted to the two groups of participants (Additional file: 1 and Additional file: 2). Healthcare workers were asked to identify measures the patients could do to reduce complications, and what information patients were supposed to have before surgery, after surgery and before discharge. Patients were asked to identify the information they needed before surgery, discharge and at home, as well as how they could contribute to reduce complications. The first author led all interviews with one of the other researchers as a moderator. The focus groups took place in quiet rooms at the hospital and the interviews lasted up to 90 min. All interviews were digitally recorded and transcribed verbatim.

### Data analysis

Inductive content analysis was used to describe the elements of surgical risks as perceived by patients and healthcare workers [27]. Four of the authors read the interviews several times. Text revealing patient safety risk were collated and divided into meaning units, which then were condensed, assigned a code and sorted into sub-categories. The entire research team discussed the sub-categories and further abstracted and reorganised them into categories. In order to identify key elements, the analysis process was retained at a descriptive category level according to Graneheim and Lundman [27]. NVivo 12 Plus software program was used to organise text and manage the data [28].

## Results

Four categories were identified throughout the analysis: pre-operative information, pre-operative preparations, post-operative information, post-operative plans and follow-up. These four categories represent the phases of information delivery, preparations and follow up. The four categories each had subcategories containing several of assigned codes, which could be identified as possible key elements for a future checklist (Table 2). However, it was also evident through our findings that the patients needed repeated information from healthcare workers and were struggling with remembering information and understanding its importance. Generally, we did not ask the healthcare workers or patients about the need for a checklist, but they both raised a need for a memory aid and clearly were positive to the idea of having patient checklists. The healthcare workers claimed that a patient checklist tool could be designed to encourage the patients to ask for information or give the healthcare staff any important information that may prevent complications or surgical cancelations. One nurse said:

**Table 2:** Key risk elements for a patient surgical checklist

CATEGORIES	SUB-CATEGORY	CODES	EXAMPLE
Pre-operative information	Contact	Direct phone number Ask questions Clarify information Write down information	***Nurse;*** *“That they have a number they can ring when they get back to them self after being informed about the surgery. Maybe it could be written a place that they get proper information under admission. I believe 90% of the patients do not remember anything.* ***Patient;*** *“Before the operation I had no idea who to contact. I got referred here and there and suddenly I got a letter informing me about an operation”.*
	Medication safety	Lack of medication lists Updated medication list at general practitioners office Little insight in own medication Identify anticoagulants with their general practitioners Learn their medications	***Surgeon;*** *“I don’t believe we always ask if they use anticoagulants and they don’t always understand that it is anticoagulants they are using. Patients often relate to the medication name and misunderstandings can often happen”.* ***Patient;*** *“I wonder what kind of information the nurses have. When I came to the ward after my surgery, I was offered some pain relief and I am allergic to certain medications. Luckily I asked her because I did not recognise those tablets and asked what they were and it turned out I was allergic to them”.*
	Health status	Ensure correct treatment Diabetes High blood pressure Cardio-Vascular disease Chronic diseases Non Healing wounds Regular control at the general practitioners office Test for multi resistance bacteria	***Nurse*;** *“I am thinking about our own health. Say, that someone is overweight; they might have diabetes that are not under control. That the general practitioners consider these things before the patients comes in for an operation.”* ***Nurse;*** *“We have talked a lot about it in our ward. I believe it is very important because some patients come in a such a bad state. So I think the elective patients should consider own health and the general practitioners should be involved earlier and help them”.*
	Optimizing health	Patients responsibility Contact with general practitioner Inform patients Exercise Stop smoking Stop drinking/drugs Nutritional status	***Surgeon;*** *“We know that it’s documented that if you quit smoking the chances of complications reduce, but there is now culture for informing patients about it.* ***Nurse;*** *“Most patients sit down in a chair and stay there, when they get informed about their surgery. I wish they could contact their general practitioner and ask how much activity they can have before surgery so they do not become passive. Because the whole thing is to optimize the patient health to prevent complications”.*
	Dental status	Regular dental checks Recommend to check dental status Poor dental status Infections due to dental status Extraction of teeth day before surgery	***Surgeon;*** *“I remember we had a patient who had to remove half of her teeth before we could operate. I believe people don’t understand how important it is that their teeth are well kept”.* ***Surgeon;*** *“one patient had an old rote canal and at the bottom of the tooth there was a little thing that they had not manage to remove. He had to go to a specialist and it was not possible to get it done the week before the surgery”.*
	Read information	Patient need for encouragement Accurate information Don’t use google	***Nurse;*** *“I know there is a lot of the patients who don’t read the information given to them”* ***Patient;*** *“When you receive all the papers before your surgery, it was too much I had no energy to read it all”*
Pre-operative preparations	Preparations 2 weeks before surgery	Type of surgery Time of surgery Bring close family/friend to information meeting Clarify when to stop anticoagulants Fill out required forms	***Patient;*** *“I should have stopped my blood thinners 2 days before my surgery, no one asked me so I stopped them the day before because I remembered it from my last operation, but it was too late”.* ***Ward doctor;*** *“When you arrive at the hospital to the information meeting it is so important that the patients bring an up to date medication list so we know what kind of medication they are using and we don’t have to wonder if they are using anticoagulants. Yes that they bring an updated list maybe this can be one of the preparations the patients need to do before their surgery”.*
	Inform your surgical ward	Patient forget to inform about important information Don’t think it’s important Other medical investigations Cold our infections just before surgery	***Patient;*** *“If you use any form for medications or need something I believe we as patients’ needs to take some responsibility to inform before surgery. Something can happen and if you have not informed about it before your surgery it is kind of your fault”.* ***Nurse;*** *“Or they actually are under investigation of other diagnoses, they have to let us know or their operation might have to be cancelled”.* ***Other Nurse:*** *“or if they get sick with throat infections and can’t be operated on”.*
	Plan your discharge	Length of hospitalisation Discharged before expected Prolonged hospitalisation due to not having someone at home Home care Aids/bandages/medication Planed discharge safer at home	***Nurse;*** *“the patients are very interested in the practical things before they come in, but we have to focus on the things that has to be ready before they get here and what they want after their surgery are they considering rehabilitation or do they live alone?”* ***Patient; “****You are going to be reduced and it is wise to have someone that can look after you at home and then you have the chance to inform the nurses that you are alone and might need an extra night in the hospital”.*
	On admission to hospital	Need for a checklist on important info Patients can check and request missing info Remove rings, necklaces, piercings When to stop eating and drinking Pre surgery shower routines Allergies Updated medication list Natural medications or nutritional supplements Are you informed about expected pain	***Patient;*** *“It was own times on drinking clear fluids, I remember it from last time I had surgery. I was told that I could drink clear fluids until a few hours before my surgery. This time I did not receive any information so I was a bit unsure when I should stop eating and drinking”.* ***Ward Doctor;*** *“We do have a journal system that is supposed to be updated with patients’ current medicines but it’s not always updated. We often use old admission notes because the patients don’t have anything with them and the referral dose not usually contain a medication list”.*
	Just before surgery	Are operational are marked correctly? Avoid getting cold Ask surgical team to use safe surgery	***Patient;*** *“The surgical team was ready and they started to look for the marked surgical site which was not there. They got a bit quiet and then they asked control questions before the operation started”.*
Post-operative information	Preventions and complications	Information about complications Often unsure at home What is normal or not What to do in an emergency Special considerations	***Surgeon;*** *“It is very important that patients contact us if they experiences complications and that they adhere restrictions”.* ***Patient;*** *“I thought this does not feel normal and I was walking around thinking Oh my god this is not good. I called the ward all the time because it was so swollen and warm. Oh my god this is not right but it apparently was all normal”.*
	Restrictions and activity	When to start exercising Stayed in bed for weeks Confusion about restrictions	***Nurse;*** *“Patients own efforts in relations to mobilisation and what they can do themselves to reduce hospitalisation time after their operation”.* ***Nurse;*** *“At the same time it is this about training, how much can they do, because they are so scared that they will damage something or do too much. But it is important that they exercise and don’t sit down”.*
	Medication safety	Start new medications Restart medications Don’t remember information Rushed information Need a checklist Ask for missing information Medication side effects New medication list	***Surgeon;*** *“it is very important to inform the patients. I believe it is the core reason for them taking their medicine and understanding their disease and that they contact their general practitioners”.* ***Patient;*** *“I believe the most important thing the doctor did was to line up all my medications. Some of them I knew from before and some I did not know. I explained to me very clearly, what each medicine was for and how long I was going to use them. This was very useful for me especially when you take 5–6 different medicines it is easy to mix them up”.*
	Pain relief	Taking too little Taking too much Regular usage When to stop What to do if still in pain	***Patient;*** *“It is easy to forget when you are laying there and then suddenly you have to ouch!! You are in your own world and suddenly it is too painful and you take double the amount of pain relief you should”.* ***Nurse;*** *“And it is reductions of pain relief, many patients have used large doses over longer time”.*
	Stomach functions	Often experiences stomach pains Constipation Prevention/medication Worried something is wrong	***Surgeon;*** *“The day after it always take some time before the stomach functions work again. This is not a problem if the patients are informed about it”.* ***Patient;*** *“I had problems with my stomach and I had to ask. I was informed that it was normal, I did not know that and it would have been good to know”.*
Post-operative plans and follow-up	Further care	Wound care Removal of sutures Other treatment Test results Sick certificate When can I shower; Whom to contact for questions	***Nurse;*** *“Further plan, times for things, how to treat the wound, showering, precautions and whom to contact”.* ***Patient;*** *“I showered with the bandage and did not change it. I looked if there was something yellow on it because I was told to. There was some wound discharge on it the first days which made me unsure”.*
	Appointments	Expected time and date Referral to other specialities What to do if not received	***Nurse;*** *“It is not always patients feel a responsibility to enquire about missing follow-up appointments. They have to take some responsibility too”.* ***Patient;*** *“kind of trust that you will get an appointment and you don’t really think about it anymore. But of course if you had a checklist or something you would go Oh I haven’t got my appointment”.*

*“I feel that sometimes information slip and the surgery are cancelled, most likely it is because of missing information and lack of communication”.*

In what follows, we present the four main categories and subcategories in detail, including representative quotes (translated from Norwegian) within each one.

### Pre-surgical information

Patients expressed a need for a contact phone number to the surgical ward to call if important issues arose. Further, they also requested to be informed about writing down any non-urgent questions they might have and bring them along to the hospital, as a memory aid.

Both healthcare workers and patients indicated that obtaining correct medications are a major problem. Lack of an updated medical list caused a lot of frustration and time-consuming work for the physicians admitting the patients to surgery and in worst cases surgery had to be postponed or even cancelled. Often the physician had to use medication lists from an earlier admission because the patients had little or no overview over their own medications. They also experienced that patients were often unaware if they used anticoagulants and patients had not stopped all these medications. One patient expressed this as follows:

*“We are not the experts here, how can I know what my medications contain? Everything on the medication package is “Greek” to me.”*

Healthcare workers suggested that if the patients were encouraged to learn their medications’ names, how they look, when to take them, and learn what the medications are for before surgery, this could reduce potential errors. In addition, they stated the patients needed to contact their general practitioner if there had been changes on the medication list that was not updated, as well as help to identify if they are using anticoagulant medications.

Healthcare workers expressed the importance of assessing the patients’ health status before surgery clearly. If they had diabetes, hypertension, cardiac or vascular diseases, non-healing wounds or other chronic health issues not having been controlled for the last months, they should contact their general practitioner to evaluate current treatment. One ward doctor said that:

*“It is a common problem that a chronic disease is often not under control before surgery, which could potential cause complications and prolonged hospitalisation.”*

Furthermore, if patients had been abroad for the last 12 months and had received dental, medical treatment, or been hospitalised or worked in a hospital or clinic, healthcare workers expressed the need for information about the importance of taking bacterial swabs for multi-resitant bacteria, in accordance with national regulations.

Patients and healthcare workers agreed that the patients themselves could take more responsibility in optimising their own health before surgery, in cooperation with their general practitioners. Patients stated that they were not aware that improving their lifestyle before their surgery could significantly reduce chances of complications. A majority thought that it was too late to change their habits only months or weeks before surgery.

Patients specifically undergoing cardiac valve replacement are required to have their dental status checked before surgery. Some cardiac and cancer patients, who had not seen their dentist, experienced that teeth had to be sanitised or extracted the day before surgery. Healthcare workers all agreed that appropriate dentist consultations before surgery could potentially reduce complications. One surgeon expressed his view on this:

*“We often have to refer the patients to get teeth sanitized in-hospital on the day before surgery, I’m not sure but I believe this is not ideal if we look at reducing the chances for infections. All our patients get informed before surgery to visit their dentist but most of them don’t.”*

Patients experienced that they rarely read information before and after their surgery. They requested more emphasis on the need to read information, and to be referred to an accurate and updated information site online. They often ended up using Google to obtain information and ended up confused and scared.

### Patient’s preparations before surgery

Patients and healthcare workers expressed that the patients were often unsure about which preparations they needed to do before their surgery and that they often forgot to fill out important forms or bring along family to information meetings, as requested. Patients said that it would be very helpful to have a list were they could tick off the most important preparations. One patient exemplified the importance of bringing family along:

*“I was so glad I had my son with me when they informed about the practical things and my surgery. I could not remember anything after the meeting, but it was not a problem because my son had also gotten the important information.”*

Furthermore, healthcare workers said that they often did not receive important medical information from the patients themselves. Either the patients did not consider crucial information important and therefore did not inform healthcare workers, or they simply forgot to inform. On occasions, healthcare workers themselves forgot to ask. Healthcare workers expressed the importance of the patients informing the surgical ward if they are under other medical investigations or if they get a cold or an infection the last week before planned surgery. In addition, patients had a great desire to have the information about when to stop eating and drinking, before their surgery – on a checklist, even though the healthcare workers mentioned this to the patients several times before their surgery. Both these points were agreed upon to potentially prevent delays or cancellation of surgery.

Healthcare workers frequently mentioned the importance of the patients having planned for their own discharge before surgery. At admission, patients often expected to be hospitalised for a longer period than planned. Many of the patients were dissatisfied when discharged earlier than expected, as theyhad not prepared for someone to stay with them the first night at home. Patients who were alone had to stay hospitalised longer because they had forgotten to organise to have someone with them the first day.

Preparations for home care is also part of the discharge planning and healthcare workers stated that the patients should be encouraged to evaluate and plan for their own need for home care or aids before admission. Patients who were well informed beforehand and had planned their hospital discharge felt more prepared for their surgery.

Healthcare workers also expressed the importance of preparing the patients for what they need to be aware of on the day of surgery. They wanted the patients to be encouraged to inform staff if the operation site was not marked or if they got a cold before surgery, to prevent surgery on the wrong side or other complications.

### Post-surgical patient information

Information about possible post-operative complications was mentioned in the interviews several times. Patients said that they often became unsure when at home, about what to expect and how to distinguish normal reactions from development of a complication. Patients and healthcare workers experienced that this caused numerous phone calls to the wards and patients travelling to and from the hospital. Some of the patients had experienced post-surgical bleedings and other complications where they had become seriously and acutely ill. Patients and their family members felt unprepared and unsure what to do in an emergency.

When it came to restrictions and activities, several patients were unsure about when they could start going for walks and exercise. Some had stayed in bed for weeks, others had been active from day one after their surgery. There was also confusion about when and how much weight they could lift after surgery, as exemplified by a patient:

*“It was difficult for me to know when I could start to lift and how much I could lift. I was worried that I could cause damages to myself and it was especially difficult because I have a little toddler at home.”*

Furthermore, patients who had to start on new medications or restart medications said that they mostly were only informed orally about their medications. In addition, they said that the information was often rushed with important points not being understood or remembered. One patient illustrated this:

“*I did not know that I could not take warfarin and ibuprofen together. My wife just said ibuprofen is much better for pain than paracetamol and I took ibuprofen for over a month before I was aware of it.”*

Pain relief was problematic for some of the patients even with written instructions on the packaging. Some patients had taken too much pain relief while others had taken too little. Healthcare workers stated that information on the importance of regular use and avoiding overuse had been provided before and after the surgery. However, it is still one of the most common questions received from patients after discharge:

*“I was in agony the second night at home. I took the pain relief I was prescribed, but it did not help. I tried calling the ward to ask for help, but no one could give me an answer. I just wished I had been informed before discharge that I could have increased my dosage, and the importance of taking your pain relief regularly.”*

According to healthcare workers many patients experience stomach pains and constipations after their surgery, and this was a common reason for the patients to call the ward. Patients were often worried that something had gone wrong even when they had been informed about the issue before and after their surgery.

### Post-surgical plans and follow-up

Both healthcare workers and patients mentioned further plans and follow-up before discharge. Patients with more complex surgery and cancer patients expressed a great need for information about this, and who to contact in different situations. This was identified as a risk factor through the patient interviews. The patients who were informed on further plans and follow-ups were much less anxious and nervous than those who were unsure on the next steps in their treatment process. Patients who did not receive their follow-up appointment at the time of discharge became unsure when to expect appointments and some had even slipped through the hospital system and been forgotten about. One healthcare worker interviewed stated:

*“It happens sometimes, that some patients fall out of the system and they do not receive their control appointment and that is very regrettable and can give serious consequences”.*

## Discussion

We identified categories and a broad spectrum of pre- and post-surgical sub-categories and codes. Our findings highlight risk elements where increased patient involvement can potentially prevent complications throughout the surgical pathway. Our main findings reflect patients’ related safety concerns and the warrant of a surgical patient safety checklist; these findings were supported by healthcare workers. The key sub-categories and codes may stimulate the patients to ask safety related questions, enhancing patient interactions with healthcare workers, optimising patients’ health before and after surgery to reduce health risks, and empowering the patients to request missing information and to be aware of medication safety, and of further care and follow-up appointments. These are all elements to be considered and incorporated into a patient safety checklist for surgical patients.

Our findings indicate that patients would like to use a safety checklist. In fact, they explicitly wanted to name it a checklist. Patients’ ability to absorb all the information provided by healthcare workers is weakened by stress and being in a vulnerable position [29]. Checklists may help focus on the most critical parts and enhance communication, as previously shown with the WHO Surgical Safety Checklist aimed at providers [21]. The patients wanted a tool to help them prepare for surgery and to remember important information when interacting with healthcare workers. A review of patient involvement in safety behaviour found that intra- and interpersonal and cultural relations between healthcare workers and patients might stimulate or limit patients’ involvement in safety [18]. By developing patients’ surgical safety checklists based on our findings, we could stimulate an interpersonal and cultural relation between the healthcare workers and patients, and encourage patient involvement. A patients’ surgical checklist might also prevent errors and reduce complications either when used alone or together with existing surgical complication prevention programs.

Combining patient’s surgical safety checklist with existing programs such as the ERAS program might improve patient’s compliance to the program as well as further reduce complications, and hospitalisation time. Several surgical complication prevention programs are based on providers giving information and patients adhering to the programs [6, 7, 13–15, 19]. The aim for a patient’s surgical safety checklist is to encourage patients to take more responsibility for their own safety, by ensuring that they have received and understood the information provided to them as well as helping them to prepare before and after surgery.

Our findings on health and personal care optimisation are in line with today’s recommendations to prevent complications by improving patient information and preparations before surgery [3, 24]. Patients are rarely informed in a timely manner about the benefits of lifestyle changes [6] and most patients participating in this study believed it was too late to change lifestyle weeks before surgery. If patients optimise their own health before surgery by exercising, improving nutritional status, or discontinuing smoking, alcohol and other substances they can reduce complications [6, 7, 30, 31]. A major study on orthopaedic patients found that ceasing smoking six to eight weeks before surgery could reduce overall complications from 56 to 18% [32]. Other studies on alcohol misuse and nutritional status have also found fewer complications if these issues are addressed six to eight weeks before surgery [6, 33]. Screening for multi-resistant bacteria, quitting smoking, treatment of chronic diseases, nutritional status, perioperative showering and body temperature and wound care after surgery are other risk elements we found, which is in line with today’s recommended key actions for patients to help prevent surgical site infections [34, 35]. Patient checklists containing elements taking into account such factors could be of great benefit.

We also identified medication safety as one of the major problems related to patient safety before and after surgery. This is also recognised as a challenge in several studies and by the WHO [14, 36, 37]. Including medication in a surgical safety checklist can help patients to be more aware of their medications and to guide them to ask the right questions to the healthcare workers before surgery and discharge which is coherent with WHO’s five moments for Medication Safety [14] and recent literature [37]. Patient checklists with elements on medications might involve the patients more in improving medication safety and reducing adverse drug events and medication errors before surgery and discharge.

The purpose of a surgical patient safety checklist is not to replace any existing educational material or clinical data but to help the patient get a better overview on the important information and preparation before and after surgery, and to serve as a communication tool. A surgical patient safety checklist might help the patients being more active in preventing errors and complications [3, 16, 24].

### Limitations and strengths

Initial focus group interviews of patients’ personal surgical experiences and healthcare workers’ expertise and knowledge are the recommended step in development of a patient checklist [24]. A limitation for this study would be that respondents only represented some surgical specialities and came from a small number of departments and institutions. Saturation in the data was achieved and no new categories emerged in the latest focus group interviews [25]. Strengths of this study are that the patients interviewed covered a large number of surgical procedures and there has been a multi-disciplinary team involved throughout the whole checklist content identification. Our findings were also in line with the current recommendations from WHO and other experts regarding involving patients in their own surgical safety [2, 13, 14, 34].

### Implications for practice and future research

Patients will be challenged and need to get more involved in safety regarding their own surgical care throughout the surgical pathway. When patients become better informed and prepared for their surgery, communication with healthcare workers should improve. Patients may consult general practitioners more often prior to surgery and less after surgery. Based on our findings a surgical checklist for patients is possible to be developed, but it will need to be designed, validated and tested before examining its effect.

## Conclusion

A wide range of risk elements have been outlined in this study, which could be the content of a patient surgical safety checklist. It is evident that patients need help with remembering information and important preparations before and after surgery that can reduce complications and unwanted errors. Based on the identified risk elements it should be possible to develop patient’s surgical checklists based on our findings.

## Supplementary information

**Additional file 1: **Focus group interview guide patients

**Additional file 2: **Focus group interview guide healthcare workers

## Data Availability

The dataset used and/or analysed during this study are available in English from corresponding author on reasonable request.

## Abbreviations

COREQ: Consolidated criteria for reporting qualitative research
ENT: Ear, neck, throat
ERAS: Prehabilitation and enhanced recovery after surgery
REC West: The regional committee for medical and health research ethics of the western Norway
SD: Standard deviations
SURPASS: Surgical patient safety system
WHO: The World Health Organisation
