# Supplementary material for: Patients’ and healthcare workers’ recommendations for a surgical patient safety checklist – a qualitative study
Source: BMC Health Serv Res. 2020 Jan 16;20:43. doi: 10.1186/s12913-020-4888-1 (PMC6966861; doi:10.1186/s12913-020-4888-1)
Supplement: Supplementary file 1 — Additional file 1: Focus group interview guide patients [file 12913_2020_4888_MOESM1_ESM.docx]

**Focus group interview guide patients**

**Organisation and implementation of the interviews**

- One person (first author) will perform all focus group interviews and one of the other three researcher will act as a moderator. One interviewer and one moderator participate in the focus group interviews. The interviews will follow the guide below with an inductive approach to the research question. It will focus on identifying risk areas to examine events and complications related to the surgical pathway.
- The ideal participant number in each interview would be 6 to 8 participants. The participants should have had surgery within one of the five included wards, and it should not be more than 2 months since their surgery.
- An information letter will be given to the patients with time and place for the interview. The participants will get a friendly reminder about time and place by text message 3 days before the interview if they agree to it.
- The participants will meet 5 min before the interview.
- Traveling cost to and from the interview will be covered.
- The participants should live within an hour drive, or have a control appointment at the hospital the same day if they live further away.
- Nursing home patients or patients with communication problems are excluded.
- Each interview will last up to 90 minutes. Current literature recommends that a focus group interview should last from 45-90 min, longer interviews are often not productive and it turns in to a burden for the participants
- The Moderator has the responsibility of keeping the time and taking field notes.
- Other preparations; we will serve coffee and tea, and fruit. We need pens, paper and recording equipment.
- Rooms has to be reserved.
- Piloting of the interviews guide will be performed before the interviews.

**Brief aims of research and focus group interviews**

The introduction of WHO’s Surgical Safety Checklist and the SURgical Patient Safety System has shown to reduce complications, morbidity and mortality, and reducing hospitalization time.^1 2^ More interventions are needed to reduce preventable surgical complications with 25% (National goal). The next step is to develop patient’s surgical safety checklists. To involve patients in the surgical pathway can be understood as a type of health promoting work and a more holistic approach to patient treatment. Patient involvement in the surgical pathway can provide a better quality assurance of that the patients have received and understood important information. An Active patient participation in the development of patients own surgical safety checklists will most likely better prepared the patient for the surgical pathway. It is shown that well planned discharge can prevent unwanted hospital readmissions.^3^ Increased patient involvement is seen as crucial to give right and secure treatment and so that the patients can have better control over their own treatment.

| **Interview steps** | **Details** |
| --- | --- |
| **Opening** | - Welcome and thank you all for participating in this focus group interview. - We need to hear your thoughts and experiences about your surgery. The reasoning is that we will use your information to develop a checklist to surgical patients. - Provide written information **(Consent signed at the point of recruiting).** - Inform the participants why they are asked to participate in this project. - Inform that the interview is recorded, anonymised, transcribed and stored securely (research server). - We have limited time so if you move away from the area examined, I will interrupt. This is not because I don’t want to hear what you have to say, but because it is important that we finish the interview to have enough information to develop the patient’s own checklists. |
| **Guidelines** | - No answer is wrong, you are allowed to have different opinions - Please, turn of the sound on your mobile phone, if you have to answer leave the room and return as soon as possible - Researcher will ask the questions, moderator will guide the discussions. - Talk to each other. - One person speaks at the time. - If there is information you do not want to talk about in the group, you can inform us after the interview in person. |
| **Group demographics (participant 1-8, start recording)** | - Recording is started and we begin with a presentation round.   Gender:  1_____2_____3______4_____5_____6_____7_____8____  Age:  1____ 2____ 3_____ 4_____5_____6_____7_____ 8_____  Type of operation  1______2_____3_____4_____5_____6______7_____8____ |
| **Introduction question** | 1. In relation to your latest surgery, can you tell us shortly about your experiences before surgery, after surgery and after being discharged |
| **Inductive discussion triggers**  **-Information -** | 1. **Core trigger: what is important for you to be informed about before surgery?** 2. Additional trigger; Can you say which of these points mentioned are most important for you? 3. Did you miss any information before your surgery? 4. If yes, what kind of information did you miss? 5. Did this have any importance for you? Disadvantages, problems 6. Did you contact the hospital before your operation? If yes, what did you call for? |
| **After surgery (still hospitalised, preparation for discharged)** | 1. **Core trigger: What information is important for you before being discharged?** 2. Additional trigger; Can you say why this is important for you? 3. Was there any information missing before your discharge? 4. If yes, what information did you miss? 5. Did this have any importance for you? Disadvantages, problems? 6. Did you have any questions regarding your discharge while you were hospitalised? |
| **After discharge** | 1. **Core trigger:** **What was important for you have information about at home in relation to your surgery?** 2. Additional trigger; Can you say which of these points mentioned are most important for you? 3. Did you miss any information after our discharge? 4. If yes, what did you miss? 5. Did this have any impact on you? Disadvantages, problems 6. Did you have to contact the hospital after discharge? If yes, why did you contact them? 7. Do you have any thoughts about what you can do to prevent complications before surgery and after? 8. We will analyse the information you have given us and use it to develop a patient’s checklist. Is it right to call this a checklist for patients? Or do you have other suggestions? |
| **Ending** | 1. Summarise the relevant findings through the interview. Is there anything we have forgotten or is there something that needs to be added? |

**References:**

1. Haynes AB, Weiser TG, Berry WR, et al. A Surgical Safety Checklist to Reduce Morbidity and Mortality in a Global Population. *New England Journal of Medicine* 2009;360(5):491-99. doi: doi:10.1056/NEJMsa0810119

2. de Vries EN, Prins HA, Crolla RM, et al. Effect of a comprehensive surgical safety system on patient outcomes. *New England Journal of Medicine* 2010;363(20):1928-37. doi: 10.1056/NEJMsa0911535

3. Hesselink G, Zegers M, Vernooij-Dassen M, et al. Improving patient discharge and reducing hospital readmissions by using Intervention Mapping. *BMC Health Services Research* 2014;14(1):1-11. doi: 10.1186/1472-6963-14-389
