# Supplementary material for: Patients’ and healthcare workers’ recommendations for a surgical patient safety checklist – a qualitative study
Source: BMC Health Serv Res. 2020 Jan 16;20:43. doi: 10.1186/s12913-020-4888-1 (PMC6966861; doi:10.1186/s12913-020-4888-1)
Supplement: Supplementary file 2 — Additional file 2: Focus group interview guide healthcare workers [file 12913_2020_4888_MOESM2_ESM.docx]

**Focus group interview guide Healthcare workers**

**Organisation and implementation of the interviews**

- One person (first author) will perform all focus group interviews and one of the other three researcher will act as a moderator. One interviewer and one moderator participate in the focus group interviews. The interviews will follow the guide below with an inductive approach to the research question. It will focus on identifying risk areas to examine events and complications related to the surgical pathway.
- The ideal participant number in each interview would be 6 to 8 participants. The participants has to be employed at one of the requited ward. It is desirable with 3 registered nurses, 1-2 ward doctor, 1-2 surgeon and one secretary/patient coordinator.
- The participants will get a friendly reminder about time and place by text message or email 3 days before the interview.
- The participants will meet 5 min before the interview.
- The participants will be interviewed within working hours.
- Each interview will last up to 90 minutes. Current literature recommends that a focus group interview should last from 45-90 min, longer interviews are often not productive and it turns in to a burden for the participants
- The Moderator has the responsibility of keeping the time and taking field notes.
- Other preparations; we will serve coffee and tea, and fruit. We need pens, paper and recording equipment.
- Rooms has to be reserved.
- Piloting of the interviews guide will be performed before the interview

**Brief aims of research and focus group interviews**

The introduction of WHO’s Surgical Safety Checklist and the SURgical Patient Safety System has shown to reduce complications, morbidity and mortality, and reducing hospitalization time.^1 2^ More interventions are needed to reduce preventable surgical complications with 25% (National goal). The next step is to develop patient’s surgical safety checklists. To involve patients in the surgical pathway can be understood as a type of health promoting work and a more holistic approach to patient treatment. Patient involvement in the surgical pathway can provide a better quality assurance of that the patients have received and understood important information. An active patient participation in the development of patients own surgical safety checklists will most likely better prepared the patient for the surgical pathway. It is shown that well planned discharge can prevent unwanted hospital readmissions.^3^ Increased patient involvement is seen as crucial to give right and secure treatment and so that the patients can have better control over their own treatment.

| **Interview steps** | **Details** |
| --- | --- |
| **Opening** | - Welcome and thank you all for participating in this focus group interview. - We need to hear your thoughts and experiences about the patient’s role in the surgical pathway. - Providing consent forms before the interview. - Inform about the aim and what the results are going to be used for; We aim to identify the most important risk areas that can be used to develop patients own surgical checklists. - Inform the participants why they are asked to participate in this project – they are employed within one of the 5 recruited surgical wards. - Inform that the interview is recorded, anonymised, transcribed and stored securely. (research server) |
| **Guidelines** | - No answer is wrong, you are allowed to have different opinions. - One person speaks at the time. - Please turn of the sound on your mobile phone, if you have to answer leave the room and return as soon as possible. - Researcher will ask the questions, moderator will guide the discussions. - Talk to each other. - If there is information you don’t want to talk about in the group, you can inform us after the interview. |
| **Gruppens bakgrunn (informant 1-6, Start opptak)** | - Recording is started and we begin with a presentation round.   Gender:  1_______2_______3________4_______5_______6_______  Age:  1_______2_______3________4_______5_______6_______  Profession:  1_______2_______3________4_______5_______6_______  Experience in years:  1_______2_______3________4_______5_______6_______ |
| **Inductive discussion triggers**  **-Information -** | Intro/statement: **This project seek to identifying risk areas of complications and for patients to be involved in reducing complication risks.**   1. What can the patients contribute with to reduce complications? And how? |
| **Inductive discussion triggers**  **-Information – before surgery** | 1. The interview is divided into 3 parts, before admission, discharge and after discharge; 2. Core trigger: **What do you think is the most important points that patients need to now before surgery to avoid complications?** 3. Additional trigger: Information before surgery, medications, diagnoses, complications. 4. Have any of you experienced that a patient was not prepared for surgery? Can you explain? 5. Does the patients ring before their surgery? And what do they ask? 6. What kind of written information do you give to the patients?      1. Do you believe the information the patient get is sufficient? If not what is missing? |
| **After surgery (still hospitalised, preparation for discharged)** | 1. Core trigger: **What are the most important issues the patients have to be aware about before discharge?** 2. Additional trigger: medications, diagnoses, complications? 3. Have you ever experienced that the patients had to stay hospitalised of missing information/preparations? Can you tell me more about this? 4. What do the patients request information about before discharge?      1. What kind of information routines do you have before discharge? Does the patient get any written information before discharge? 2. Do you believe this is enough? If not what is missing? |
|  | 1. Core trigger: **What is the most important things for the patients to know after discharge?** 2. Additional trigger: medications, diagnoses, complications etc? 3. Have you experienced readmission because of missing information? Please explain? 4. What does the patient contact the hospital about after discharge? 5. Do you believe patients own surgical checklists can reduce complications? And when do you think the patients need to receive the surgical checklist? |
| **Ending** | 1. Summarise the relevant findings through the interview. Is there anything we have forgotten or is there something that needs to be added? |

# References

1. Haynes A, Weiser T, Berry W, Lipsitz S, Breizat A, Dellinger E, Herbosa T, Joseph S, Kibatala P, Lapitan M, Merry A, Moorthy K, Reznick R, Taylor B, Gawande A. A Surgical Safety Checklist to Reduce Morbidity and Mortality in a Global Population. *NEJM* 2009; 360:491-499.

2. Haugen AS, Søfteland E, Almeland SK, Sevdalis N, Vonen B, Eide GE, Nortvedt MW, Harthug S. Effect of the World Health Organization Checklist on Patient Outcomes: A Stepped Wedge Cluster Randomized Controlled Trial. *Annals of Surgery* 2015; 261(5):821-828.

3. Nilssen H. Program for pasienttryggleik i Helse Vest 2013. Accessed 06.13, 2016.

4. Solberg R. Stortingsmelding 11 (2015-2016). Nasjonal helse- og sykehusplan (2016-2019). *In* omsorgsdepartementet H-o, ed. Regjeringen.no: Regjeringen, 2015. pp. 146.

5. Solberg R. Kvalitet og pasientsikkerhet 2013. *In* omsorgsdepartementet Ho, ed., Vol. Stortingmelding nr. 11 (2014-2015). Regjeringen.no, 2014.

6. de Vries EN, Ramrattan MA, Smorenburg SM, Gouma DJ, Boermeester MA. The incidence and nature of in-hospital adverse events: a systematic review. *Qual Saf Health Care* 2008; 17(3):216-23.

7. Holmboe O, ØA. B. Pasienterfaringer med norske sykehus i 2014. Lokale rapporter og resultater. . 2015.

8. Doyle C, Lennox L, Bell D. A systematic review of evidence on the links between patient experience and clinical safety and effectiveness. *BMJ Open* 2013; 3(1).

9. Kripalani S, Theobald CN, Anctil B, EE. V. Reducing Hospital Readmission Rates: Current Strategies and Future Directions. *Annu Rev Med* 2014; 65:14.

10. Hesselink G, Zegers M, Vernooij-Dassen M, Barach P, Kalkman C, Flink M, Ön G, Olsson M, Bergenbrant S, Orrego C, Suñol R, Toccafondi G, Venneri F, Dudzik-Urbaniak E, Kutryba B, Schoonhoven L, Wollersheim H. Improving patient discharge and reducing hospital readmissions by using Intervention Mapping. *BMC Health Services Research* 2014; 14(1):1-11.
